# Pulmonary fate and consequences of transferrin-functionalized gold nanoparticles

**DOI:** 10.7150/ntno.47734

**Published:** 2021-03-05

**Authors:** Nagarjun Venkata Konduru, Karen Velasco-Alzate, Sitaramaraju Adduri, Kyryl Zagorovsky, Daysi Diaz-Diestra, Faisalina Fisol, Marcelo Sanches, Harrison Ndetan, Joseph David Brain, Ramon Morales Molina

**Affiliations:** 1 Department of Cellular and Molecular Biology, University of Texas Health Science Center at Tyler, 11937 U.S. Hwy 271, Tyler, TX 75708, USA.; 2 Luna Nanotech Inc., 439 University Avenue, 5th Floor, Toronto, ON, Canada M5G 1Y8.; 3 Center for Nanotechnology and Nanotoxicology, Harvard T.H. Chan School of Public Health, 665 Huntington Avenue, Boston, MA 02115, USA.; 4 Department of Biostatistics, University of Texas Health Science Center at Tyler, 11937 U.S. Hwy 271, Tyler, TX 75708, USA.

**Keywords:** protein corona, alveolar macrophage, lung clearance, organ retention, primary human monocyte-derived macrophages

## Abstract

Surface functionalization of nanoparticles (NPs) may alter their biological interactions such as uptake by alveolar macrophages (AMs). Pulmonary delivery of gold NPs (Au NPs) has theranostic potential due to their optoelectronic properties, minimal alveoli to blood translocation, and possibility of specific cell targeting. Here, we examined whether coating Au NPs with transferrin alters their protein corona, uptake by macrophages, and pulmonary translocation.

**Methods:** Rats were intratracheally instilled with transferrin-coated Au NPs (Tf-Au NPs) or polyethylene glycol-coated Au NPs (PEG-Au NPs). AMs were collected and processed for quantitation of Au cell uptake using ICP-MS and electron microscopy. Au retention in the lungs and other organs was also determined. The uptake of fluorescently labeled Tf-Au NPs and PEG-Au NPs by monocyte-derived human macrophages was also evaluated *in vitro*.

**Results:** We showed that Tf-Au NPs were endocytosed by AMs and were retained in the lungs to a greater extent than PEG-Au NPs. Both Au NPs acquired similar protein coronas after incubation in rat broncho-alveolar lavage fluid (BALf). The translocation of Au from both NPs to other organs was less than 0.5% of the instilled dose. Transferrin coating enhanced the uptake of Au NPs by primary monocyte-derived human macrophages.

**Conclusions:** We report that coating of NP surface with transferrin can target them to rat AMs and human monocyte-derived macrophages. NP functionalization with transferrin may enhance NP-based therapeutic strategies for lung diseases.

## Introduction

Surface functionalization of nanoparticles (NPs) and resulting physicochemical properties play critical roles in the effects and biokinetics of NPs following systemic administration 1-3. For example, the surface coating of intravenously injected NPs has been shown to reduce opsonization and phagocytosis by macrophages with direct access to the blood, thus increasing particle circulation time 4, 5. However, the effects of surface characteristics on the biokinetics of NPs delivered to the lungs have not been studied. The protein corona that forms on NPs upon interaction with lung lining fluids has been shown to alter the fate of both naive and surface-functionalized NPs 6. However, the nature of the encounter of NPs with lung lining fluid is poorly characterized but is critical in determining the subsequent fate of and responses to NPs 7. Several studies have examined the role of the protein corona on biokinetics of functionalized NPs after intravenous delivery. However, very little is known about the nature of NP-biomolecular interactions in the alveoli despite growing interest in pulmonary delivery of nanocarriers for local drug delivery.

In a recent study, we measured AM uptake of albumin- and citrate-coated Au NPs recovered from rats 24 h after IT-instillation 8. We found that the percentage of AMs with internalized Au NPs was not different between rats instilled with albumin-coated compared with citrate-coated Au NPs. We found that both albumin- and citrate-coated Au NPs acquired similar protein coronas, a plausible explanation for the lack of differences in their AM uptake and lung clearance 8. However, AMs that had taken up albumin-coated Au NPs had fewer endosomes with densely packed NPs than AMs with citrate-coated Au NPs.

Surface functionalization of NPs with polyethylene glycol (PEG) is a common strategy to enhance drug encapsulation efficacy and prolong the circulating half-life and drug release 9. PEGylation also reduces the uptake of NPs by phagocytic cells such as macrophages 10. In light of the extensive use of PEGylation of NPs for drug delivery, it is important to understand how PEG affects the interactions of NPs with proteins present in the lung lining fluid as well as the fate and clearance of NPs in the lungs. It has been shown that the translocation and biodistribution of gold NPs delivered to the lungs intratracheally (IT) were not different between PEG- and citrate-coated gold NPs 3. However, the biodistribution and excretion patterns of these two NP types were different after intravenous administration 3.

Studies have shown that functionalization of NPs with carbohydrates and glycoproteins increases the site-specific delivery of drugs into AMs 11-13. Transferrin (Tf) is a naturally abundant glycoprotein in the lung lining fluid that enhances iron transport and is endocytosed via the Tf-receptor (TfR). Targeted drug delivery of NPs functionalized with Tf has been widely used for cancer cell targeting because tumor cells overexpress TfR 14. Overexpression of TfR has been reported in liver, breast, lung and colon cancer cells 15. AMs internalize carbohydrate-based agents via mannose 16, 17 or mannose-containing receptors such as TfR. The transferrin receptor has glycosylation sites and contains high-mannose oligosaccharides 18. Information on the clearance kinetics and biodistribution of Tf-functionalized NPs is currently lacking. In this study, we studied whether coating Au-NPs with transferrin would influence their interaction with AMs when delivered into the lungs. We instilled rats with Tf-functionalized Au NPs (Tf-Au NPs) and PEG-functionalized (PEG-Au NPs), and examined their uptake by AMs, lung clearance, and translocation to secondary organs. We also examined the formation of hard protein corona, the layer of proteins irreversibly bound on to the surface of AuNPs, when incubated in rat lung lining fluid since this corona might alter the fate of Au NPs *in vivo*. Finally, we examined whether functionalization of Au NPs with Tf altered their uptake by monocyte-derived human macrophages *in vitro*.

## Methods

### Synthesis and physicochemical characterization of Au NPs

**Figure 1:**
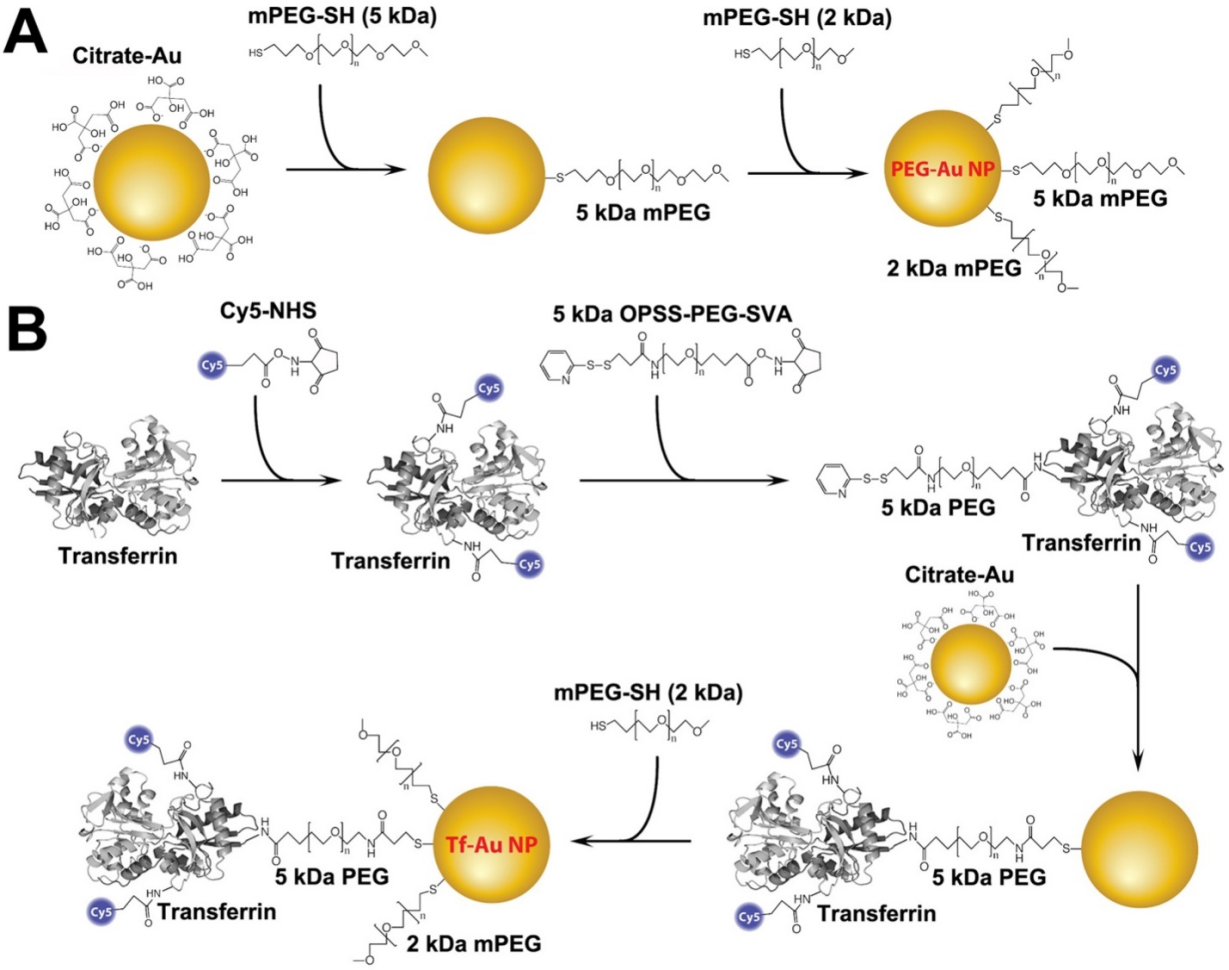
Schematic representation of the functionalization of PEG- and transferrin-Au NPs. **A**. PEG-Au NP synthesis. Five kDa methoxy-terminated PEG molecules were adsorbed onto the surface of 15 nm Au NPs through their terminal thiol groups. **B**. Transferrin-Au NP synthesis. Transferrin protein was first conjugated to a Cy5 fluorophore, and then to an amine-reactive 5 kDa PEG spacer with a terminal protected orthopyridyl disulfide (OPSS) thiol group. This construct was then adsorbed onto Au NP surface through the formation of a thiol-gold bond. The surface was further stabilized by backfilling it with a thiol-terminated 2 kDa methoxy PEG.

Tf-Au NPs and PEG-Au NPs were synthesized using a citrate reduction method and functionalized with the appropriate ligands following the protocols shown in **Figure 1**. PEG-Au NPs were synthesized by adsorbing 5 kDa methoxy-terminated PEG molecules onto the surface of 15 nm Au NPs through the interaction with their terminal thiol groups. For Tf-Au NP synthesis, transferrin protein was first conjugated to a Cy5 fluorophore, then to an amine-reactive 5 kDa PEG spacer with a terminal protected orthopyridyl disulfide (OPSS) thiol group. This construct was then adsorbed onto Au NP surfaces through the formation of a thiol-gold bond. The surface was further stabilized by backfilling it with a thiol-terminated 2 kDa methoxy PEG. Absorbance and fluorescence profiles of both Au NPs were measured using a UV-1601PC UV Vis (Mandel, Guelph, Canada) and a FluoroMax-3 fluorometer (Horiba, Kyoto, Japan), respectively. Electron microscopy was performed using a FEI Tecnai 20 (Thermo Fisher Scientific, Waltham, MA). The hydrodynamic diameter (D_H_), polydispersity index (PdI), and zeta potential (ζ) of each suspension were measured using a Zetasizer Nano-ZS (Malvern Instruments, Worcestershire, UK).

### Characterization of NPs after incubation in BALf: protein corona and dynamic light scattering analyses

A total of 9 ml pooled BALf from 3 rats (3 ml/rat) was centrifuged at 350 × g to remove cells. Then, Tf-Au and PEG-Au NPs (200 μL of 1 mg/mL) were incubated in the 3 mL BALf for 1 hour at 37^°^C. We chose a 1-hour incubation since corona formation in the lungs occurs soon after the particles interact with the alveolar lining and because those early events are relevant to their fate in the lungs 19, 20. After incubation, the NP suspensions were centrifuged for 10 min at 14,500 × g. The resulting pellets were washed in deionized (DI) water three times. The use of DI water avoided the potential interaction of hydrophobic phospholipid components of the BALf with the corona proteins. The pellets containing NPs with 'hard corona' were suspended in 20 μL of DI water. One set was used for DLS analyses and the other set for protein corona composition analyses.

The NP pellets for protein corona analyses were suspended in 20 μL of DI water to which 10 μL of 4× Laemmli sample buffer was added. The sample was then vortexed and heated to 95 °C for 7 min. The sample was then cooled to room temperature and 6 μL of mixed solution (57 μL Laemmli and 3 μL β-mercaptoethanol) was added to 18 μL of each sample. The samples were then loaded onto a gel and electrophoresed. The proteins were visualized by 1-D SDS-PAGE in combination with Coomassie staining. The protein corona experiment was performed twice (n=6 rats).

Distinct bands from 2 gels were excised and then digested using a modified in-gel trypsin digestion procedure 21. Peptides were extracted and then dried in a Speed-Vac (~1 h) at 4 °C. Samples were analyzed at the Harvard Medical School Taplin Mass Spectrometry facility (Boston, MA) as previously described 22.

### Animals

The Harvard Medical Area Institutional Animal Care and Use Committee (Boston, MA) approved the animal protocols in this study. All procedures were performed in accordance with relevant guidelines for humane treatment of animals. Twenty-four male Wistar Han rats (8 weeks old) were purchased from Charles River Laboratories (Wilmington, MA). They were housed in standard microisolator cages under controlled conditions of temperature, humidity, and light at the Harvard Center for Comparative Medicine. The animals were provided with commercial chow (PicoLab Rodent Diet 5053, Framingham, MA) and reverse-osmosis purified water *ad libitum*. All animals were acclimatized in the facility for 7 days before the start of each experiment.

### Assessment of *in vivo* uptake of NPs by rat alveolar macrophages

Tf-Au and PEG-Au NP suspensions were instilled in rats (n=3 per NP) at a dose of 1 mg/kg and concentration of 0.67 mg/ml. The instilled volume was 1.5 ml/kg body weight. Twenty four hours later, the rats were sacrificed and their lungs were lavaged, as described previously 23. An aliquot of BAL cells from each NP group were processed for ICP-MS quantitation of Au. Another aliquot was cytocentrifuged and fixed in 2.5% glutaraldehyde in HEPES buffer, pH 7.4. The pellets were processed for electron microscopy. Uptake of NPs by cells was analyzed in a JEOL 1400 transmission electron microscope (JEOL USA, Inc., Peabody, MA). Random micrographs from each rat were scored for the presence of internalized NPs in each macrophage. Macrophage uptake was scored as +, ++, or +++ when 1-2, 3-4, or ≥ 5 particle-containing phagosomes were observed in randomly selected electron micrographs, respectively. A total of 200 macrophages per NP type were scored.

### Pharmacokinetics of Au after intratracheal instillation of Au NPs

Fifty microliters of 1 mg/ml NP suspension were added to a calculated volume of sterile distilled water to achieve a volume dose of 1.5 ml/kg body weight. The effects of physiologic buffered saline (PBS) and water instilled into rat lungs showed no significant differences in BAL parameters except myeloperoxidase (Figure S2). Although considered unsafe for intratracheal instillation, the use of water as the suspending medium for the NPs was to avoid (a) NP surface interactions with constituents of PBS, and (b) potential resultant hypertonicity with added NPs. The mean weight of rats was 275 ± 10 g. Before dosing, each rat was anesthetized with isoflurane (Piramal Healthcare, Bethlehem, PA). The NP mass doses were based on calculated NP concentrations measured by UV-Vis spectroscopy. However, based on ICP-MS analysis, the final Au dose was 23 μg Au/rat (Tf-Au NPs) and 30 μg Au/rat (PEG-Au NPs). The NP suspension was intratracheally instilled into the lungs of rats (n=6 rats per NP). Then, six rats from each group were euthanized at 24 hours post-dosing. They were anesthetized with vaporized isoflurane and exsanguinated via the abdominal aorta. The lungs, tracheobronchial lymph nodes, spleen, liver, feces, and urine were collected for Au analysis using ICP-MS. Data were expressed as percentage of the administered Au dose retained in each organ/sample.

### Isolation of human peripheral blood monocytes and macrophage differentiation

Forty-five ml of blood samples from healthy adult donors were collected in complete RPMI 1640 (Cat# SH3025501, Cytiva, Marlborough, MA) (1% Sodium Pyruvate, 10% Fetal Bovine Serum and 1% Pen/Strep, GIBCO). Institutional review board at the University of Texas Health Science Center at Tyler (UTHSCT) approved these studies. Mononuclear cells were separated by Ficoll gradient method (1:3) by centrifuging cells at 500 x g (1600 rpm for 25 min). The cells were resuspended and washed twice with RPMI 1640, and then centrifuged at 400 x g (1350 rpm for 8 min). The monocytes were purified by positive selection using the CD14 MicroBeads (Miltenyi Biotech) according to manufacturer's recommendations. The cells were counted and resuspended in complete RPMI 1640 with 10% inactivated human serum. Cells were seeded on flacon 12-well plates (Fisher scientific) at a density of 0.5x10^6^ cells/well and incubated for 2 hours. After this time, the non-adherent cells were removed by soft mechanical lavage with complete RPMI 1640 containing 10% inactivated human serum inactivated. The monocytes were then cultivated in 2 ml of complete RPMI 1640 with 10% inactivated human serum supplemented with 5 ng/ml of GM-CSF, which was changed every third day until maturation and full differentiation of macrophages. Two to four wells were treated separately with complete RPMI 1640 with inactivated 10% human serum without the growth factor for comparative evaluation of monocyte differentiation with that of GM-CSF stimulated monocytes.

### Confirmation of human monocytes differentiation to macrophages by flow-cytometry

To evaluate the degree of differentiation of monocytes to M1 macrophages after 7 days of stimulation with GM-CSF, the macrophages and monocytes (unstimulated cells) were removed with scraper, washed with FACS buffer (1X PBS with 3% FBS) and surface marked with anti-Human CD14- PE (Clone M5E2, Biolegend, San Diego, CA), anti-Human CD11b-APC (Clone ICRF44, Biolegend, Cat# 301310), Anti-Human CD1a-BV711 (Clone HI149, Biolegend, Cat# 300139) and Anti-Human CD163-APC-Cy7 (Clone GHI/61, Biolegend, Cat# 333621) at 4 °C for 30 min. The cells were checked for CD14 purity by flow-cytometry in BD LSRFortessa^TM^ X-20 (BD Biosciences).

### Analysis of uptake of Au NPs by human monocyte-derived macrophages

Monocytes differentiated into macrophages were washed with 1X PBS and incubated with 5 µg/ml Au NPs (labeled with Alexa Fluor 647) in RPMI 1640 without serum for 0, 1 and 4 hours at 37 °C in a 5% CO_2_ atmosphere. After each incubation time, the supernatant was collected and frozen at -80 °C for future cytokine analysis (Eve Technologies, Alberta, Canada). The remaining cells were washed with PBS and then gently removed by scraping. The cells were resuspended in FACS buffer, centrifuged at 400 × g (1400 rpm for 5 min) and the pellet was resuspended in 200 ul of FACS buffer and kept on ice before analysis. The flow cytometry data on uptake of fluorescently labeled NPs by cells were acquired in BD LSRFortessa^TM^ X-20 (BD Biosciences, San Jose, CA) and analyzed by FlowJo software (TreeStar, Ashland, OR). The analysis included the determination of % of fluorescent positive cells (% of cells with Au NPs) and the Mean Fluorescence Intensity (MFI) determining the relative amount the Au NPs inside of the macrophages.

To confirm if NP internalization was dependent on an active endocytotic process, 5 µg/ml Au NPs were added to the differentiated cells in RPMI 1640 without serum on ice and then were incubated at 4 °C with 5 µg/ml Au NPs in RPMI 1640 without serum for 1 hour. Furthermore, in order to define the significant pathway involved in the internalization of PEG- and Tf-Au NPs, we performed experiments using inhibitors of clathrin-mediated endocytosis. Macrophages were pre-treated for 30 min with the mixture of 100 µM chlorpromazine hydrochloride (CPZ) and 250 µM monodansylcadaverine (MDC), the inhibitors of clathrin-mediated endocytosis 24, and then exposed to the 5 µg/ml AuNPs labeled with Alexa flour 647 in RPMI 1640 medium without serum for 4 hours. All samples were then prepared for uptake analysis by flow-cytometry as described above.

### Hyperspectral imaging of gold nanoparticles

Gold nanoparticles inside monocyte-derived human macrophages were imaged using a CytoViva® enhanced dark-field microscope (CytoViva, Auburn, AL, USA). The presence of gold particles were evaluated in macrophages at 60× and 100× magnifications. Au NPs were diluted to 250 μg/mL in water, loaded onto clean glass microscope slides, and cover slipped in an aqueous permount mounting medium (Fisher Sci, Waltham, MA). Then mean spectral profiles for particles were generated. These profiles were created using pixels with an intensity greater than 2000 (Pixelfly PCI camera, Harvard Bioscience, Holliston, MA). To determine a mean spectrum from gold nanoparticles inside cells, a minimum of 1000 pixels of Au NP in the cells were collected to form a region of interest. This spectrum was then compared with the suspended gold particle control and the unexposed cells without Au NPs.

### Statistical analyses

The Au tissue retention data were analyzed using multivariate analysis of variance (MANOVA) followed by Tukey *post hoc* tests using SAS Statistical Analysis Software (SAS Institute, Cary, NC). The data for ICP-MS, macrophage uptake of Au NPs and flow cytometry data were analyzed using Student's t test.

## Results

### Synthesis and physicochemical characterization of gold nanoparticles

**Figure 2:**
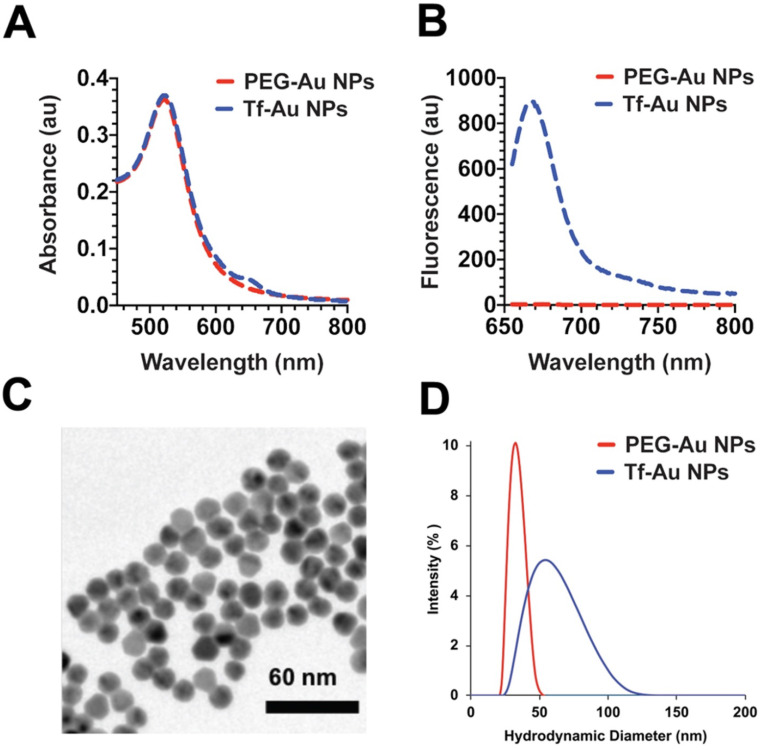
Characterization of PEG-Au and Tf-Au NPs. **A**. UV and Vis absorbance profiles of the nanoparticle constructs. **B**. Fluorescence spectrum of PEG-Au and Tf-Au nanoparticles. **C**. Transmission electron micrograph of PEG Au NPs. **D**. Size distribution of PEG-Au and Tf-Au NPs suspended in distilled water. Hydrodynamic diameters were analyzed by dynamic light scattering.

The Au NPs used in this study were prepared as illustrated in Figure 1. For PEG-Au NP, 15 nm citrate-coated gold nanoparticles were reacted with a thiol and methoxy-terminated 5 kDa PEG. PEG was adsorbed onto the nanoparticle surface through the formation of a thiol-gold coordinate bond (**Figure 1A**). For the synthesis of PEG-Au NPs, fluorescently labeled with Alexa Fluor 647, a mixture of amine-terminated 2 kDa PEG-SH (15%) and methoxy-terminated 5 kDa PEG-SH (85%) was used instead. Following the conjugation of PEG onto the nanoparticle surface, amine-terminated 2 kDa PEG on the nanoparticle surface was then reacted with amine-reactive Alexa Fluor 647. For Tf-Au NPs, transferrin protein was first labeled with a Cy5 fluorophore then covalently conjugated to an amine-reactive 5 kDa PEG spacer. The opposite end of the spacer contained a protected orthopyridyl disulfide (OPSS) thiol group, which was then used to adsorb transferrin-PEG onto the surface of 15 nm NPs through the formation of a thiol-gold bond. The surface density of transferrin was 15 ± 3 Tf molecules/NP. Tf-Au NPs were further stabilized by backfilling the surface with a 2 kDa methoxy PEG, which was adsorbed onto the NPs through a terminal thiol group (**Figure 1B**). The backfill with 2 kDa PEG is at 5 PEG/nm^2^. The physicochemical characteristics of PEG-Au and Tf-Au NPs were confirmed by measuring their absorbance and fluorescence spectra, dynamic light scattering, and images from transmission electron microscopy (Figure 2). Absorbance profile peaking around 520 nm for both nanoparticles was characteristic of 15 nm Au NPs (**Figure 2A**). Presence of fluorescently labeled Tf was confirmed by a small absorbance peak near 650 nm and a fluorescence peak at 665 nm (**Figure 2B**). The individual particles were spherical with diameter of 15 nm (**Figure 2C**). Prior to use in experiments, PEG-Au and Tf-Au NPs were diluted to 0.67 mg/ml with additional sterile PBS. These final suspensions were used in all *in vivo* experiments and for determination of surface charge and hydrodynamic diameters (D_H_). Using dynamic light scattering (DLS), we observed mean D_H_ of 32.7 and 54.6 nm, polydispersity index (PDI) of 0.02 and 0.15, and zeta potentials of -6.1 mV and -11.3 mV for PEG-Au NPs and Tf-Au NPs, respectively (**Table 1**). The hydrodynamic size distributions of the two Au NPs are shown in **Figure 2D**. From these measurements, we found that the average transferrin density on NP surface was 15 ± 3 Tf molecules per NP. The sizes of NPs measured by DLS are consistent with sizes reported in previous publications 25, 26.

### Characterization of the protein corona on gold nanoparticles

**Figure 3:**
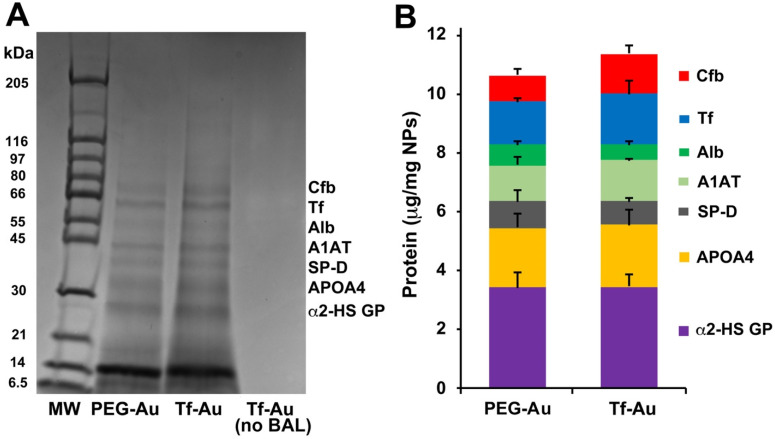
Analysis of protein corona on Au NPs after incubation with BALf. **A**. 1-D gel electrophoresis and mass spectrometry of NP-bound rat BAL proteins. A representative gel from one experiment is shown. Seven proteins identified by LC-MS are indicated on the right. The molecular weights (kDa) of reference proteins are shown in lane MW. **B**. Quantification of specific corona proteins extracted from Au NPs. Data are mean ± SE µg/mg NPs (n=3 rats per NP).

As the corona may modulate the overall NP biokinetics, we first examined the composition of acquired protein corona on the two Au NPs after incubation in bronchoalveolar lavage fluid (BALf). We also determined whether the acquired corona influences NP agglomerate size and zeta potential. The hydrodynamic diameter of PEG-Au NPs changed from 32.7 to 158 nm while Tf-Au NPs increased from 54.6 to a much larger size (> 1 µm) (**Table 1**). We analyzed the composition of the acquired NP protein corona using methods described previously 27. We performed 1-D SDS-PAGE and Coomassie staining of total proteins detached from the NPs to visualize the corona components on gel bands. The proteins contained in the excised gel bands were identified in an LTQ Orbitrap Velos Pro ion-trap mass spectrometer. The stained SDS-PAGE gel and the relative amounts of identified proteins after NPs were incubated with BALf are shown in **Figure 3A** and** B**. We found no significant differences in the corona composition between Tf- and PEG-Au NPs. We found that in both cases, the corona primarily contained proteins such as complement factor B, transferrin, albumin, α-1 antitrypsin, surfactant protein D, apolipoprotein A4, and α-2-HS-glycoprotein. From these experiments, we conclude that the protein corona contributes little to the agglomeration of the NPs, especially Tf-Au NPs.

### Alveolar macrophage uptake and lung retention of gold nanoparticles

As recognition, binding and phagocytosis of particles by AMs is a major determinant of lung clearance, we next determined if AM uptake of PEG- and Tf-Au NPs *in vivo* were different. At 24 h post-instillation, rat lungs were repeatedly lavaged to obtain AMs and other cells for analyses. The numbers of harvested cells, which were mostly macrophages, were not different between the two NPs (**Table 2**). The total amounts of Au analyzed by ICP-MS in lavaged cells from rats instilled with Tf-Au (1.01 ± 0.05 µg) was greater than with PEG-Au NPs (0.62 ± 0.07 µg). The amounts of Au recovered in BAL supernatant representing non-internalized Au-NPs were 1.94 µg (PEG-Au NPs) and 0.95 µg (Tf-Au NPs) (**Table 2**).

**Figure 4:**
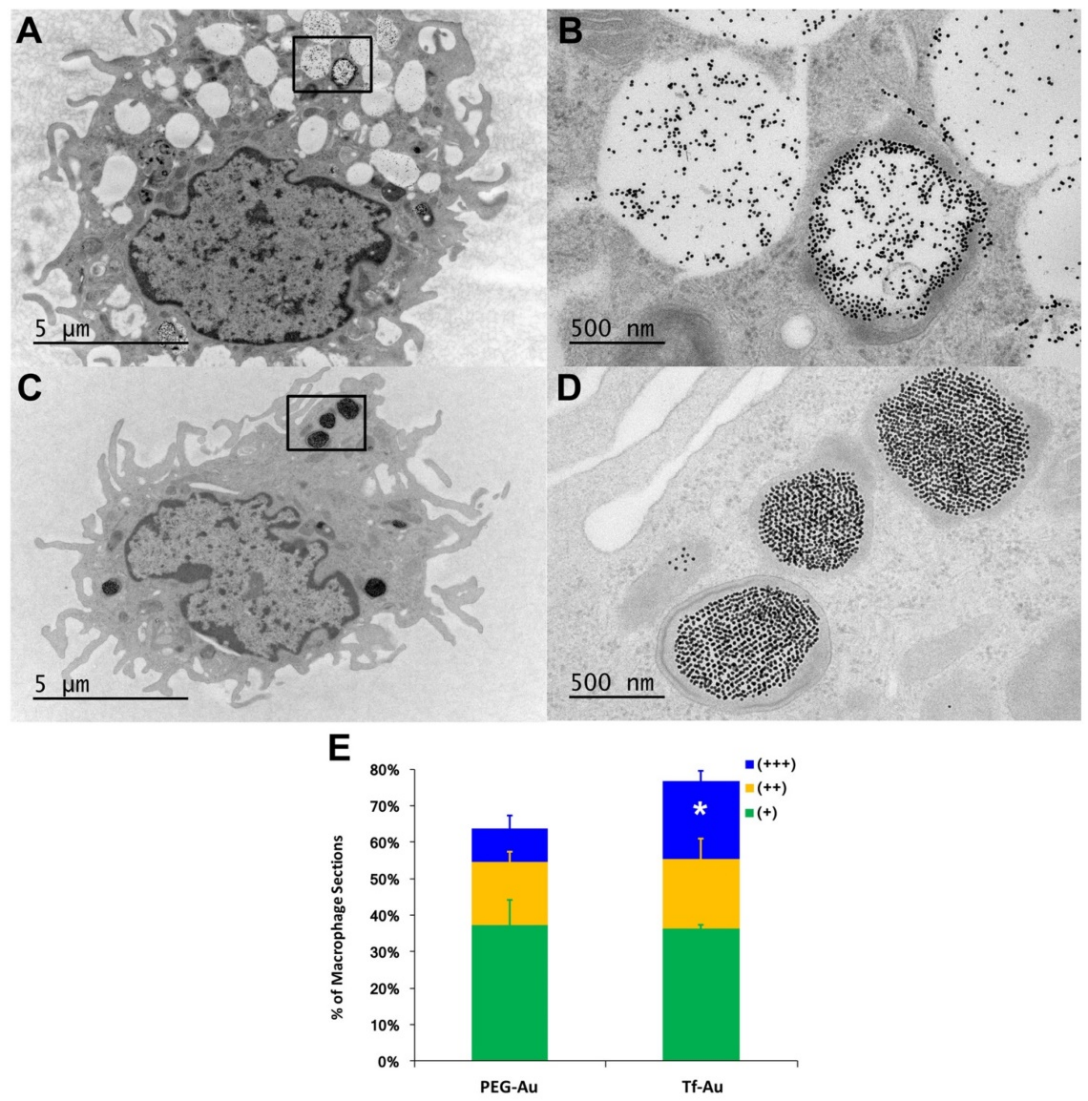
Transmission electron micrographs of lavaged cells from rats at 24 h post-instillation of NPs at a dose of 1 mg/kg body weight. Macrophage uptake of Au NPs was scored as +, ++, or +++ when 1-2, 3-4 or ≥ 5 particle-containing endosomes were observed in macrophages (n=200 cells), respectively. **A, B**. Macrophage uptake of PEG-Au NPs. Figure 4B shows higher magnification of enclosed area in 4A showing endosomes with loosely packed PEG-Au NPs. **C, D**. Macrophage uptake of Tf-Au NPs. Figure 4D shows higher magnification of enclosed area in 4C showing endosomes with densely packed Tf-Au NPs in honeycomb patterns. **E**. Morphometric analysis of macrophage uptake of NPs. A significantly higher percentage of AMs with +++ scores was observed with Tf-Au versus PEG-Au NPs. The total amount of Au measured by ICP-MS was also higher in AMs from Tf-Au NP-instilled animals. Data are mean ± SE of % of macrophages (n=3 rats per NP).

We also measured the extent and appearance of NP endocytosis by AMs recovered from BALf at 24 h post-instillation. Using TEM, we found that macrophages that had taken up Tf-Au NPs (**Figure 4C, 4D**) exhibited more endosomes with densely packed NPs compared to macrophages that had taken up PEG-Au NPs (**Figure 4A, 4B**). The total fraction of macrophage sections with internalized Tf-Au NPs was about 1.2 times higher than with PEG-Au NPs (**Figure 4E**). In addition, a greater percentage of macrophage sections contained ≥ 5 Tf-Au NP-containing phagosomes (21.2%) than with PEG-Au NPs (9.0%). The percentage of sections of AMs with internalized Tf-Au NPs was also significantly greater than with PEG-Au NPs (**Figure 4E**). These morphological observations are consistent with the quantitative differences in Au content in lavaged cells between the two NPs measured by ICP-MS.

**Figure 5:**
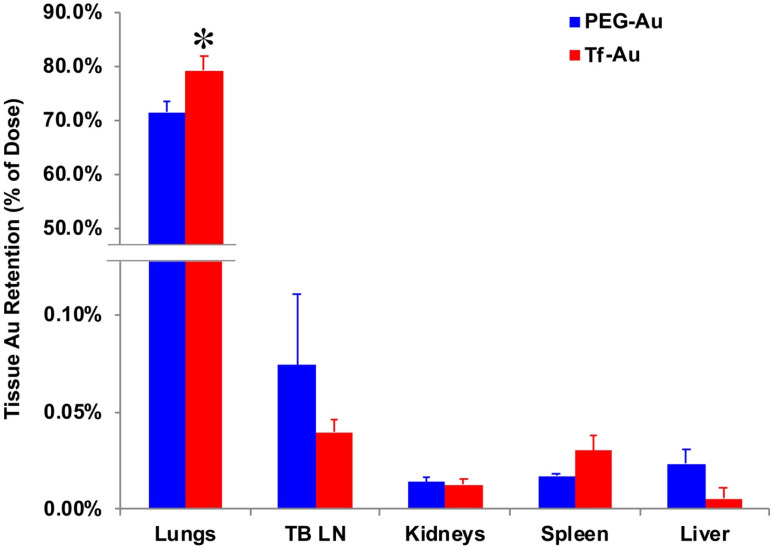
Tissue distribution of Au at 24 h after IT instillation of PEG-Au and Tf-Au NPs in rats. The bulk of measured Au was found in the lungs. Measured Au in the lungs of Tf-Au NP-instilled rats was significantly higher than in PEG-Au NP-instilled rats (p<0.05). Low percentages of instilled Au were measured in the liver, spleen, kidneys and tracheobronchial lymph nodes (TBL). Data are mean ± SE (n=6 rats per NP).

To determine the effects of transferrin coating on the lung retention of Au NPs, we IT-instilled rats with 1 mg/kg of PEG- or Tf-Au NPs. The final measured delivered doses were 23 μg Au/rat (Tf-Au NPs) and 30 μg Au/rat (PEG-Au NPs). At 24 h post-instillation, we measured the Au content in the lungs and other organs such as the liver, spleen, the major organs of the mononuclear phagocyte system, and the tracheobronchial lymph nodes using ICP-MS. All tissue Au concentration data were normalized to the instilled Au dose delivered to each rat. We observed that the amount of Au from Tf-Au NPs retained in the lungs was significantly higher (79.2% of instilled Au) than from PEG-Au NPs (71.4%) (p≤ 0.05) (**Figure 5**). Interestingly, the extrapulmonary retention of Au from both NPs did not differ. The organ retention of Au was in the order of tracheobronchial lymph nodes > spleen > liver (**Figure 5**). The excretion of Au was mostly via the feces (1.2-1.4%). Overall, these experiments indicate that Tf-Au NPs exhibited higher retention in the lungs and were taken up more efficiently by alveolar macrophages than PEG-Au NPs.

### Uptake of gold nanoparticles by primary human monocyte-derived macrophages

**Figure 6:**
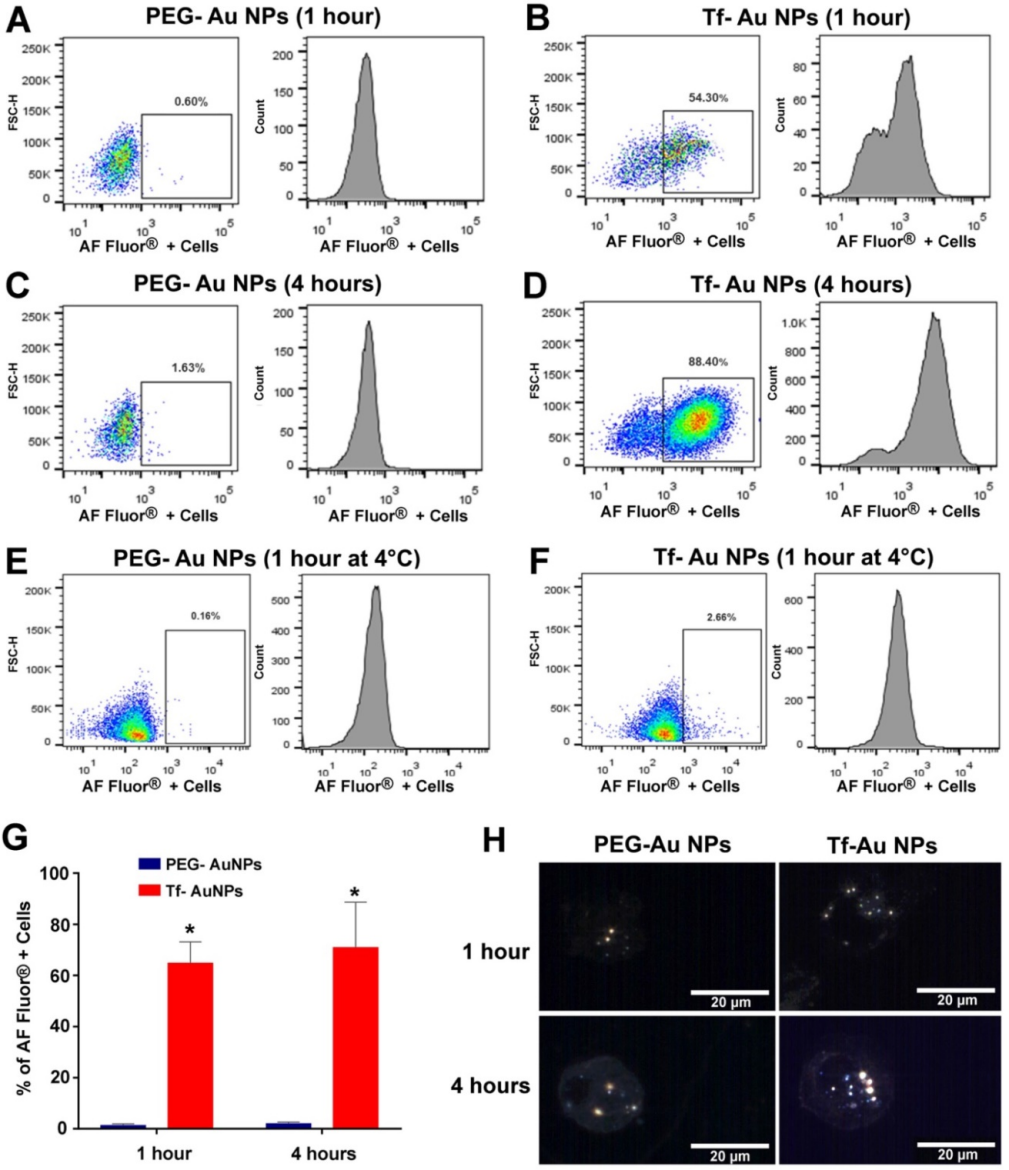
Primary human monocyte-derived macrophages recognize and internalize fluorescently labeled Tf-Au NPs but not PEG-Au NPs. Human monocyte-derived macrophages (0.5×10^6^ cells/well; n=6) were incubated with PEG-Au NPs or Tf-Au NPs for 1 hour and 4 hours. The cells were then evaluated for uptake of NPs by flow-cytometry. Flow-cytometry analysis of fluorescence positive cells assayed at 1 hour (**A, B**) and 4 hours (**C, D**) from a representative experiment are shown. **E, F**. Assessment of uptake of Au NPs by macrophages at 4°C is shown. **G**. Percentages of fluorescence positive cells for the two NP types at variable times are depicted. Cells incubated with PEG-Au NPs showed significantly lower fluorescence (data are mean ± SE, n=6; *p< 0.001). **H**. Representative hyperspectral imaging confocal micrographs of macrophages at 100X magnification. The internalized NPs appeared to aggregate within the cells.

As the focus of this study was to determine the role of Tf functionalization of NPs on their uptake by macrophages, we used primary human monocyte-derived macrophages as a model system. For quantitative assessment of uptake of NPs, we used Tf-Au and PEG-Au NPs fluorescently labeled with Alexa Fluor 647. We studied the time-course of uptake of these Au NPs for up to 4 hours using flow cytometry. Viability of the cells determined using trypan blue exclusion assay at this time point was 96%. We found that there was significant time-dependent increase in number of cells positive for fluorescently labeled Tf-Au NPs (**Figure 6A-D**). The number of FACS positive cells for Tf-Au NPs was 50- fold higher than PEG-Au NPs at 1-hour (**Figure 6G**). The increase in the number of positive cells for Tf-Au NPs was 43-fold higher than PEG-Au NPs over the 4-hour incubation time (**Figure 6G**). As the internalization of transferrin and its receptors is mediated through clathrin-mediated endocytosis, the uptake was drastically reduced for both NP types when the cells were incubated with NPs at 4 °C (**Figure 6E, 6F**). This was expected as lower temperatures slow down active cellular processes. Furthermore, we found that the internalization of the Tf-coated Au NPs was impaired when macrophages were pre-treated with inhibitors of clathrin-mediated endocytosis (Figure S1, Supplementary file).

To evaluate whether the Au NPs were internalized or just cell surface bound, hyperspectral imaging of NPs was performed focusing on the nucleus of the cell at 100× magnification. Hyperspectral imaging confirmed that internalization of the NPs dominated (**Figure 6H**). These experiments demonstrate that the transferrin functionalized AuNPs are internalized at an enhanced rate compared to PEG-AuNPs. Moreover, inhibition of the endocytotic machinery substantially reduced the endocytosis of Tf-coated Au NPs by macrophages.

## Discussion

Receptor-mediated strategies are an attractive approach for NP drug delivery. Uptake of NPs by AMs can influence their physiologic activities such as defense against intracellular pathogens and modulation of pro- and anti-inflammatory responses. The type of receptor involved in the recognition and uptake of NPs may determine subsequent responses 28. Similarly, targeting of other tissue resident macrophages is an attractive therapeutic strategy for treating diseases associated with aberrant macrophage activity. Various macrophage surface receptors, e.g., scavenger receptors, phosphatidyl serine receptors, thrombospondin receptors, integrins, and complement receptors, have been implicated in recognition and uptake of particles 29. Macrophages also express transferrin receptors (TfR) on their surface for bactericidal activity 30. As macrophages are enriched in TfR, we explored if functionalization of NPs with Tf can facilitate their uptake by rat AM and human monocyte-derived macrophages. Will doing so alter their lung clearance and translocation to extrapulmonary tissues after lung delivery?

We hypothesized that coating of Au-NPs with transferrin and their delivery into the lungs will influence their interaction with AMs and fate in the lungs. As the corona may modulate the overall NP biokinetics and biological effects, we further analyzed the protein corona formed on the two NPs after incubation in rat BAL fluid. After incubation in BALf, the aggregate sizes and magnitude of surface charges of both NP suspensions were significantly altered. Compared with the suspension in PBS, both Au types after acquiring coronas exhibited larger and more variable hydrodynamic diameter. Tf-Au NPs also formed larger agglomerates than the PEG-Au NPs. We acknowledge that the measurement of NP size in complex matrices such as lung lining fluid is difficult with the current state-of-the-art techniques. Sample preparation and NP isolation require repeated centrifugation and washing steps to remove the unbound proteins and phospholipids prior to size characterization of NPs. The *in vitro* NP size measurements may not accurately reflect the NP behavior *in vivo*. Hence, the *in vitro* data on NP sizes need to be interpreted with caution. As shown in **Figure 3**, the coronas of both Au NPs consisted of transferrin and other proteins of the lung lining fluid. Quantitative evaluation of major proteins adsorbed on the surface of both NPs revealed no differences. However, it should be noted that the lung lining fluid comprises 80-90% phospholipids in addition to proteins. Since we examined only the protein, the role of phospholipid interactions with Tf- and PEG-AuNPs and their potential contribution to NP agglomeration needs to be established. The lack of a higher amount of extracted transferrin from Tf-Au NPs can be attributed to the fact that the original transferrin was covalently conjugated to the PEG-linker on the surface of Tf-Au NPs and unlikely to contribute to the total extracted Tf corona (**Figure 3**). The acquisition of a similar corona may also be due to the necessity of methoxy-terminated PEG backfilling in both the NP types for functionalization to stabilize the NPs against charge-induced aggregation 31. The number and amounts of proteins bound to PEG- and Tf-Au NPs were greater than to albumin- and citrate-coated Au NPs reported previously 8.

As functional groups on the surface of NPs and the type of biomolecules comprising the corona significantly influence the uptake of NPs by AMs, we also evaluated the uptake of Au NPs by AMs. ICP-MS quantitation of Au content in AMs confirmed that Tf-Au NPs were taken up by AMs more readily than PEG-Au NPs (**Table 2**). Likewise, the total fraction of macrophage sections with internalized Tf-Au NPs was higher than with PEG- Au NPs and contained more particle-containing endosomes, which suggests that surface functionalization with transferrin might enhance endocytosis. Similar degrees of uptake by AMs was previously observed with citrate and albumin coating of Au NPs, where coating with the latter enhanced AM phagocytosis to the same extent as seen with transferrin coating 8. Electron micrographs revealed the presence of Tf-Au NPs in a tightly packed honeycomb-pattern cluster within endosomes of the macrophages (**Figure 4D**), correlating with a TfR-mediated endocytic mechanism where active clustering of Tf receptors precedes clathrin-coated pit initiation followed by entry into endosomes 32. In contrast, PEG- Au NPs were loosely packed in endosomes (**Figure 4B**). The data suggest that increased aggregate size and surface charge of Tf-Au NPs after protein corona acquisition *in vivo* might have contributed to the greater uptake by AMs. Pretreatment of human monocyte-derived macrophages with a mixture of inhibitors of clathrin-mediated endocytosis, chlorpromazine and monodansylcadaverine, substantially reduced the uptake of Tf-coated Au NPs by macrophages.

Our pharmacokinetic data showed that there was a significant difference in the retention of the two Au NPs in the lungs. We observed that higher amounts of Au from the Tf-Au NPs (79.2%) than from PEG-Au NPs (71.4%) remained in the lungs 24 h post-instillation despite lower delivered Tf-Au NPs (23 μg) than PEG-Au NPs (30 μg). The amount of Au from PEG-Au NPs (71.4%) was also significantly lower than from citrate-Au NPs (80.8%) shown previously 8. Interestingly, in spite of lower lung retention of PEG-Au NPs, less than 0.5% of both Au NPs translocated from the lungs to the tracheobronchial lymph nodes, kidney, liver, and spleen, consistent with the minimal translocation reported in other pharmacokinetic studies on Au NPs 8, 33. Clearance of NPs from the alveoli is a slow process involving phagocytosis of NPs by AMs followed by bioprocessing and dissolution of the soluble particles. In addition, small fraction of NPs may translocate into the lymphatics and subsequently accumulate in regional lymph nodes. As Au NPs are likely resistant to dissolution within intracellular compartments, a higher uptake of insoluble Tf-Au NPs in macrophages might have contributed to the lower lung clearance of Tf-Au NPs compared to PEG-Au NPs.

Finally, we wanted to determine whether Tf-PEG Au NPs could be recognized and internalized by other tissue resident macrophages. As peripheral blood monocytes replenish tissue-resident macrophage populations, we next carried out experiments using primary human monocyte-derived macrophages. The flow-cytometry experiments demonstrated that Tf- Au NPs were endocytosed by human macrophages more effectively than PEG- Au NPs (**Figure 6A-D, 6G**). Two membrane receptors on macrophages (TfR1 and TfR2) have been implicated in the internalization of transferrin 34. Transferrin facilitates iron uptake in cells upon binding to the receptors and is internalized by clathrin-mediated endocytic mechanism 35. In order to determine if the internalization of Au NPs was an active transport process, cells were incubated with NPs at 4 °C. The uptake of all NP types was remarkably reduced under these experimental conditions suggesting that an active endocytic process was primarily involved in the internalization of Au NPs (**Figure 6E, 6F**). Hyperspectral imaging was performed to evaluate the internalization of NPs and was in agreement with the flow cytometry data (**Figure 6H**). To be considered for theranostic and other biomedical applications, NPs should not induce adverse cytokine responses. Therefore, we measured the levels of cytokines secreted by primary human monocyte-derived macrophages upon treatment with Au NPs functionalized with either Tf or PEG only. Notably, out of the forty cytokines and chemokines analyzed, we did not observe any significant differences between cells exposed to NPs and unexposed cells up to 4 hours of incubation (Table S1, Supplementary file). However, the cytokine response to Tf-Au NPs and PEG-Au NPs at a longer duration of exposure needs to be evaluated.

## Conclusions

Our data shows that Au NPs functionalized with either PEG or transferrin bind the same proteins as they interact with lung lining fluids. However, we showed that Au NPs coated with transferrin were endocytosed by alveolar macrophages to a greater extent than PEG-Au NPs despite acquisition of similar protein coronas. Our pharmacokinetic data also show that a greater amount of Au from Tf-Au NPs was retained in the lungs than from PEG-Au NPs but translocated to extrapulmonary organs to the same degree. Interestingly, in spite of slightly greater lung retention of Tf-Au NPs, less than 0.5% of Au NPs translocated from the lungs, which is consistent with the minimal translocation reported in other pharmacokinetic studies. We also provided evidence of differential uptake of NPs functionalized with Tf compared to PEG-Au NPs in human monocyte-derived macrophages. Our study demonstrates that alteration of NP surface alters their interaction with lung macrophages and monocyte-derived macrophages, which may prolong lung retention. This can be a viable NP-based therapeutic strategy for treatment of diseases associated with dysfunctional macrophages.

## Supplementary Material

Supplementary figures and tables.

## Figures and Tables

**Table 1:** Characteristics of Au nanoparticles

	PEG-Au NPs	Tf-Au NPs
	NPs in PBS	
D_H_ (nm)	32.7	54.6
PdI	0.02	0.15
ζ (mV)	-6.1	-11.3
	**NPs with coronas in PBS (post-30 min incubation in BALf)**	
D_H_ (nm)	158	> 1000
PdI	0.29	0.89
ζ (mV)	-16.7	-15.7

PEG: polyethylene glycol; Tf: transferrin; Au-NPs: gold nanoparticles; PBS: phosphate buffered saline; D_H_: hydrodynamic diameter; PdI: polydispersity index; ζ: zeta potential; BALf: bronchoalveolar lavage fluid.

**Table 2:** Distribution of recovered Au in the lungs at 24 h post-instillation in rats

	PEG-Au NPs			Tf-Au NPs		
Total Cells (×10^6^)	4.65	±	0.79	4.24	±	0.64
Au/million cells (µg)	0.14	±	0.02	0.25	±	0.04*
Total Au in cells (µg)	0.62	±	0.07	1.01	±	0.05*
Total Au in supernatant µg)	1.94	±	0.36	0.95	±	0.19

*p≤ 0.05, Student's t test, n=3; PEG: polyethylene glycol; Tf: transferrin; Au-NPs: gold nanoparticles.

## Abbreviations

NPs: nanoparticles
AMs: alveolar macrophages
Au NPs: gold nanoparticles
Tf-Au NPs: transferrin-coated Au NPs
PEG-Au NPs: polyethylene glycol-coated Au NPs
ICP-MS: inductively coupled plasma mass spectrometry
IT: intratracheal
TfR: transferrin receptor
OPSS: orthopyridyl disulfide thiol group
D_H_: hydrodynamic diameter
DLS: dynamic light scattering
PDI: polydispersity index
BALf: bronchoalveolar lavage fluid
1-D: one dimension
SDS-PAGE: sodium dodecyl sulfate-polyacrylamide gel electrophoresis
LTQ: linear trap quadropole
TEM: transmission electron microscope
FACS: fluorescence activated cell sorting
TBL: tracheobronchial lymphnodes
DI: deionized
MANOVA: multivariate analysis of variance
SAS: Statistical Analysis System
