# Supplementary material for: Pulmonary fate and consequences of transferrin-functionalized gold nanoparticles
Source: Nanotheranostics. 2021 Mar 5;5(3):309–20. doi: 10.7150/ntno.47734 (PMC7961126; doi:10.7150/ntno.47734)
Supplement: Supplementary file 1 — Supplementary figures and tables. [file ntnov05p0309s1.pdf]

Supplementary File: Pulmonary fate and consequences of transferrin-functionalized gold nanoparticles

Nagarjun Venkata Konduru [a *], Karen Velasco-Alzate [a], Sitaramaraju Adduri [a], Kyryl Zagorovsky [b], Daysi Diaz-Diestra [c], Faisalina Fisol [c§], Marcelo Sanches [c], Harrison Ndetan [d], Joseph David Brain [c], and Ramon Morales Molina [c *]

[a] Department of Cellular and Molecular Biology, University of Texas Health Science Center at Tyler, 11937 U.S. Hwy 271, Tyler, TX 75708, USA.

[b] Luna Nanotech Inc., 439 University Avenue, 5th Floor, Toronto, ON, Canada M5G 1Y8

[c] Center for Nanotechnology and Nanotoxicology, Harvard T.H. Chan School of Public Health, 665 Huntington Avenue, Boston, MA 02115, USA.

[d] Department of Biostatistics, University of Texas Health Science Center at Tyler, 11937 U.S. Hwy 271, Tyler, TX 75708, USA.

[§]Current Address: School of Pharmaceutical Sciences, 11800 Universiti Sains Malaysia, Pulau Pinang, Malaysia.

Corresponding Author:

***** Nagarjun V. Konduru, Department of Cellular and Molecular Biology, University of Texas Health Science Center at Tyler, 11937 U.S. Hwy 271, Tyler, TX 75708, USA.

Telephone: 1-903-877-7224, Fax: 1-903-877-7316

Email: Nagarjun.KonduruVenkata@uthct.edu

1. Clathrin-mediated endocytosis of Tf-Au NPs by macrophages: In order to define the significant pathway involved in the internalization of Tf-AuNPs, we performed experiments using inhibitors of clathrin-mediated endocytosis. Macrophages were pre-treated for 30 min with the mixture of 100 μM chlorpromazine hydrochloride (CPZ) and 250 μM monodansylcadaverine (MDC), the inhibitors of clathrin-mediated endocytosis [1] , and then exposed to the 5 μg/ml AuNPs labeled with Alexa flour 647 in RPMI 1640 medium without serum for 4 hours. We found that the inhibitors CPZ and MDC efficiently reduced the internalization of Tf-AuNPs (Supplementary Figure 1), establishing that the internalization of Tf-AuNPs was predominantly via the clathrin-mediated endocytosis route. The total number of macrophages with fluorescently labeled PEG-AuNPs was less than 0.5 % even without the treatment with endocytosis inhibitors.

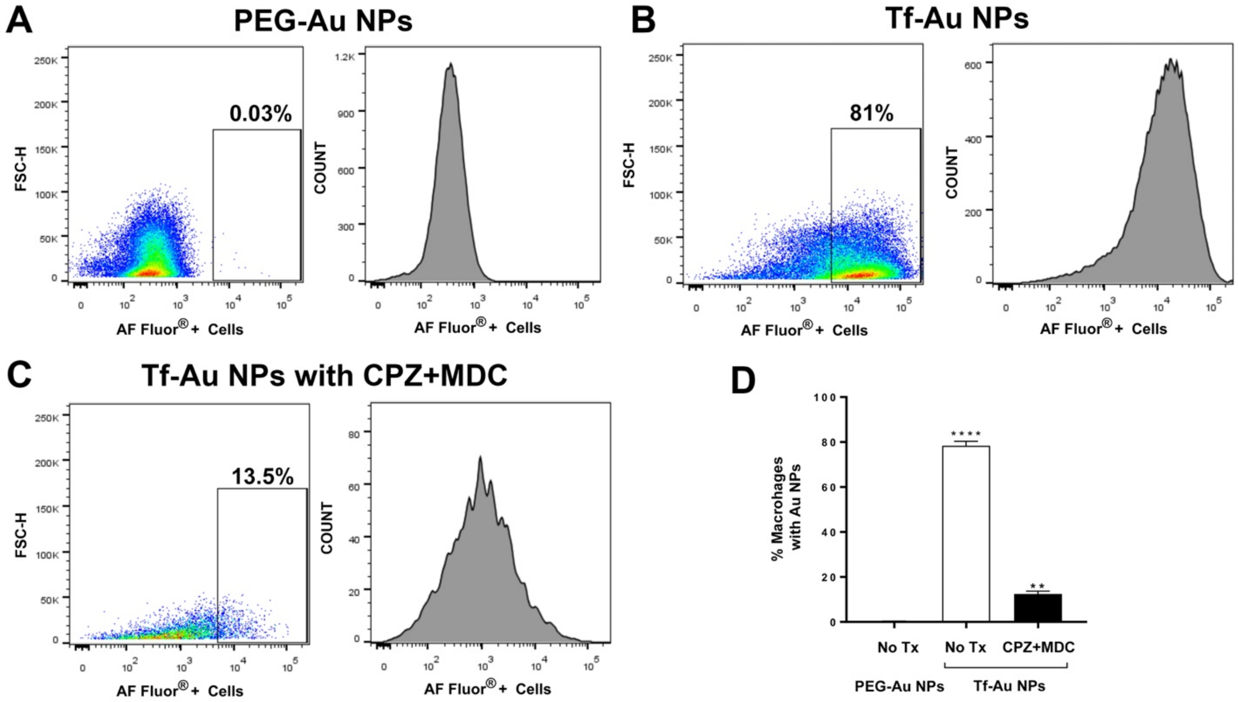

Figure S1: Flow-cytometry analysis of fluorescent positive cells assayed at 4 hours in the presence and absence of clathrin-mediated endocytosis inhibitors. Cells pre-treated with the

endocytosis inhibitors showed significantly lower mean fluorescence intensity as seen in the flow-cytometry plots. The percentage of fluorescence positive cells analyzed for internalization of Alexa Fluor-647-labeled AuNPs (data are mean ± SE, n=3; $p<0.001$).

2. Analysis of cytokines and chemokines secreted by macrophages in response to PEG- and Tf-AuNPs. Macrophages ($5x10^5$ cells/well) were incubated with 5 µg/ml of PEG-AuNPs or Tf-AuNPs in RPMI 1640 medium without serum for 4 hours. Cell culture supernatants were sent to Eve Technologies (Calgary, Alberta, Canada) for the measurement of 60 cytokines and chemokines. A Milliplex polystyrene beads (Millipore, Billerica, MA) on a Luminex platform was used for the measurement of cytokines and chemokines. The analysis shows that there were no significant differences in cytokines and chemokines secreted by macrophages up to 4 hours post-exposure to AuNPs.

Table S1. Cytokine, chemokine and growth factors measured in supernatant 4 hours after incubation of human monocyte-derived macrophages with PEG-Au or Tf-Au NPs. Data are mean ± SE pg/ml, n= 5 per NP.

Analyte	PEG-AuNPs	Tf-AuNPs	p-Value
EGF	0.83 ± 0.17	1.08 ± 0.23	0.086
Fractalkine	8.21 ± 2.32	6.05 ± 0.84	0.075
RANTES	2.46 ± 1.40	2.12 ± 1.02	0.555
G-CSF	0.80 ± 0.21	0.71 ± 0.09	0.154
GM-CSF	2.49 ± 0.62	2.72 ± 0.66	0.912
sCD40L	0.23 ± 0.08	0.24 ± 0.08	0.942
I-309	0.19 ± 0.04	0.18 ± 0.04	0.774
MCP-2	2.42 ± 0.42	2.41 ± 0.38	0.886
TRAIL	0.59 ± 0.19	0.61 ± 0.23	0.819
BCA-1	0.09 ± 0.02	0.09 ± 0.03	0.555
ENA-78	2.14 ± 0.76	3.80 ± 1.31	0.319
GROα	10.14 ± 2.42	12.25 ± 1.57	0.421
Eotaxin	2.02 ± 0.13	2.20 ± 0.33	0.164

Eotaxin-2	25.69 ± 9.54	12.16 ± 4.31	0.219
Eotaxin-3	3.07 ± 1.24	3.23 ± 1.32	0.904
IP-10	2.83 ± 1.39	2.30 ± 1.29	0.888
IFNy	0.40 ± 0.06	0.50 ± 0.153	0.125
TNF-α	16.58 ± 3.61	14.49 ± 1.77	0.277
TNF-β	0.12 ± 0.05	0.24 ± 0.07	0.509
TGF-α	0.18 ± 0.08	0.17 ± 0.07	0.901
PDGF-AA	0.49 ± 0.11	0.50 ± 0.08	0.572
PDGF-BB	2.35 ± 1.00	1.13± 0.53	0.246
VEGF-A	0.18 ± 0.09	0.50 ± 0.18	0.201
MCP-3	3.76 ± 0.63	3.96 ± 0.87	0.559
6CKine	1.48 ± 0.71	1.78 ± 0.81	0.788
MIP-1α	80.81 ± 45.11	92.78 ± 41.19	0.885
MIP-1β	18.22 ± 13.42	31.03 ± 16.24	0.762
FGF-2	18.17 ± 1.713	20.89 ± 2.62	0.598
Flt-3L	1.521 ± 0.29	1.39 ± 0.42	0.475
IL-1β	0.81 ± 0.18	0.68 ± 0.12	0.398
IL-2	0.12 ± 0.05	0.17 ± 0.08	0.494
IL-3	0.11 ± 0.04	0.16 ± 0.07	0.452
IL-4	1.58 ± 0.45	1.92 ± 0.35	0.642
IL-5	0.18 ± 0.05	0.19 ± 0.06	0.816
IL-6	0.29 ± 0.05	0.32 ± 0.06	0.643
IL-7	0.25 ± 0.08	0.23 ± 0.07	0.670
IL-8	312.80 ± 64.62	208.40 ± 40.80	0.470
IL-9	0.15 ± 0.05	0.16 ± 0.08	0.579
IL-10	0.16 ± 0.07	0.17 ± 0.08	0.811
IL-13	0.31 ± 0.03	0.36 ± 0.04	0.598
IL-12P40	0.83 ± 0.27	1.37 ± 0.26	0.930
IL-12P70	0.39 ± 0.040	0.46 ± 0.07	0.346
IL-15	0.18 ± 0.08	0.17 ± 0.07	0.871
IL-17A	0.10 ± 0.03	0.09 ± 0.04	0.616
IL-18	1.05 ± 0.65	1.12 ± 0.69	0.913
IL-20	7.76 ± 5.25	7.08 ± 2.89	0.353
IL-21	1.06 ± 0.44	1.06 ± 0.45	0.974
IL-23	8.78 ± 0.62	12.19 ± 3.95	0.011
IL-28A	0.54 ± 0.17	0.46 ± 0.19	0.789
IL-33	1.24 ± 0.43	1.30 ± 0.48	0.838
SCF	0.73 ± 0.21	0.77 ± 0.21	0.964
IFNα2	1.33 ± 0.33	1.48 ± 0.29	0.836
MDC	45.34 ± 11.03	52.29 ± 16.54	0.574
LIF	0.92 ± 0.27	0.98 ± 0.35	0.621

CTACK	0.29 ± 0.11	0.30 ± 0.13	0.793
SDF-1a+B	2.14 ± 0.76	3.80 ± 1.31	0.319
TARC	0.07 ± 0.03	0.07 ± 0.03	0.942
TPO	1.894 ± 0.63	2.16 ± 0.72	0.787
TSLP	0.54 ± 0.22	0.56 ± 0.24	0.872
MIP-1d	2.72 ± 1.12	2.87 ± 1.23	0.862

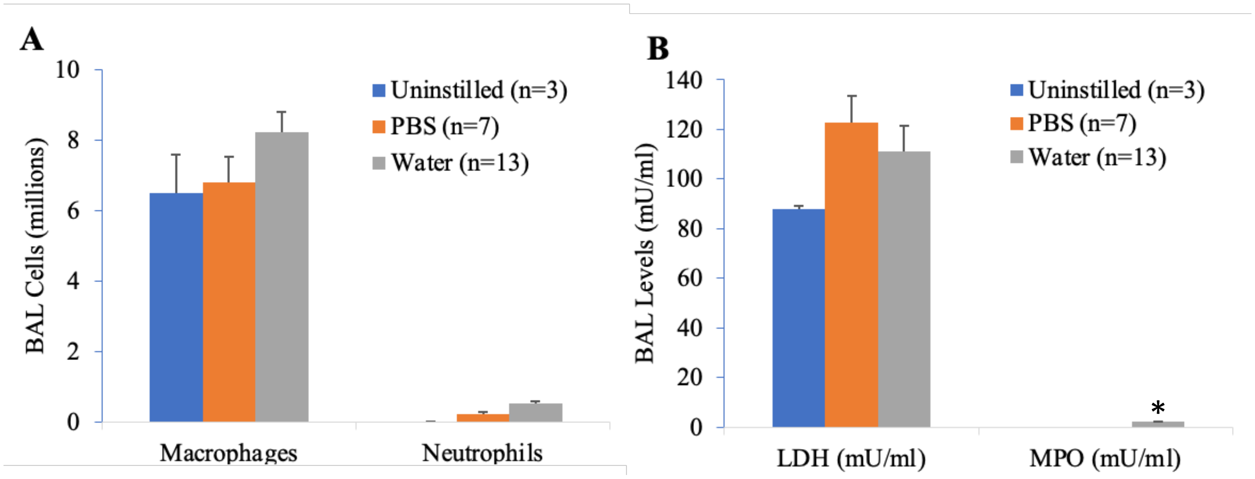

Figure S2. Cellular and biochemical parameters in bronchoalveolar lavage from rats at 24 hours post-instillation with 1.5 ml/kg sterile phosphate saline (PBS) or distilled water. Uninstilled rats served as control. **A.** Macrophages and neutrophils, and **B.** lactate dehydrogenase and myeloperoxidase activities in bronchoalveolar lavage. * $P<0.05$, PBS vs. water, Student t test.

Reference

1. Kuhn, D.A., D. Vanhecke, B. Michen, F. Blank, P. Gehr, A. Petri-Fink, and B. Rothen-Rutishauser, Different endocytotic uptake mechanisms for nanoparticles in epithelial cells and macrophages. Beilstein J Nanotechnol, 2014. **5**: p. 1625-36.
